# High diagnostic performance of plasma and cerebrospinal fluid beta‐synuclein for sporadic Creutzfeldt–Jakob disease

**DOI:** 10.1002/acn3.51873

**Published:** 2023-08-08

**Authors:** Samir Abu‐Rumeileh, Steffen Halbgebauer, Giuseppe Mario Bentivenga, Lorenzo Barba, Simone Baiardi, Andrea Mastrangelo, Patrick Oeckl, Petra Steinacker, Angela Mammana, Sabina Capellari, Markus Otto, Piero Parchi

**Affiliations:** ^1^ Department of Neurology Martin‐Luther‐University Halle‐Wittenberg Halle (Saale) Germany; ^2^ Department of Neurology Ulm University Hospital Ulm Germany; ^3^ German Center for Neurodegenerative Diseases (DZNE e.V.) Ulm Germany; ^4^ Department of Biomedical and Neuromotor Sciences (DIBINEM) University of Bologna Bologna Italy; ^5^ IRCCS Istituto delle Scienze Neurologiche di Bologna Bologna Italy

## Abstract

Beta‐synuclein is a promising cerebrospinal fluid and blood biomarker of synaptic damage. Here we analysed its accuracy in the discrimination between sporadic Creutzfeldt–Jakob disease (*n* = 150) and non‐prion rapidly progressive dementias (*n* = 106). In cerebrospinal fluid, beta‐synuclein performed better than protein 14‐3‐3 (AUC 0.95 vs. 0.89) and, to a lesser extent, than total tau (AUC 0.92). Further, the diagnostic value of plasma beta‐synuclein (AUC 0.91) outperformed that of plasma tau (AUC 0.79) and neurofilament light chain protein (AUC 0.65) and was comparable to that of cerebrospinal fluid biomarkers. Beta‐synuclein might represent the first highly accurate blood biomarker for the diagnosis of sporadic Creutzfeldt–Jakob disease.

## Introduction

The use of cerebrospinal fluid (CSF) biomarkers of neurodegeneration and prion pathology as well as brain magnetic resonance imaging (MRI) have significantly improved the diagnostic assessment of sporadic Creutzfeldt–Jakob disease (sCJD).^1^, ^2^, ^3^, ^4^ Nevertheless, sCJD diagnosis remains challenging due to the wide disease heterogeneity, including six major disease subtypes with distinct clinicopathological features and its clinical overlap with other forms of rapidly progressive dementia (RPD).^5^, ^6^ Moreover, CSF markers are not ideal for screening purposes considering the invasiveness of the lumbar puncture (LP), and reaching a high accuracy in the diagnosis using brain MRI requires a level of operator experience that may not be present at all hospitals.^4^, ^7^, ^8^

In this context, developing a highly accurate blood biomarker might present considerable advantages in terms of minimal invasiveness, wide applicability for early diagnosis, and tracking of disease progression. However, available markers, such as blood neurofilament light chain protein (NfL) and tau protein, although showing a good prognostic value,^8^, ^9^, ^10^ are of limited utility in discriminating prion disease from other RPDs.^8^, ^10^, ^11^

Recently, we have reported elevated levels of the presynaptic protein beta‐synuclein (beta‐syn) in CSF and blood of patients with prion disease as a surrogate marker of synaptic damage.^12^, ^13^, ^14^ Here, we compared CSF and plasma beta‐synuclein performance to that of classic surrogate biomarkers [CSF total tau (t‐tau), CSF 14‐3‐3 protein, plasma tau, and plasma NfL] in the discrimination between sCJD and non‐prion RPD forms in the clinical setting and evaluated beta‐syn prognostic value in sCJD.

## Methods

### Case classification

We analysed retrospectively CSF and plasma samples from patients with RPD submitted to the neuropathology laboratory at the Institute of Neurological Sciences of Bologna between 2004 and 2022. Detailed inclusion criteria are reported in supplementary methods. We included 150 patients with sCJD and 106 participants representative of the most common etiologies of non‐prion RPD.^6^ The sCJD group encompassed 82 definite and 68 probable cases with a significant number of patients from the three most prevalent sCJD subtypes [MM(V)1, VV2, and MV2K].^1^, ^5^ For the analysis according to the molecular subtype, we merged the subjects with definite sCJD with those with a probable diagnosis and a high level of certainty for a given subtype (supplementary methods).^4^, ^15^ Diagnoses of non‐prion RPD cases are provided in Table S1.

Survival was defined as the time (in months) from LP or blood collection to (i) death or (ii) akinetic mutism if life‐extending treatments (e.g., enteral/parenteral nutrition, tracheostomy) were performed.^10^

The study was conducted according to the revised Declaration of Helsinki and Good Clinical Practice guidelines and approved by the ethics committee “Area Vasta Emilia Centro” (approval number AVEC:18025, 113/2018/OSS/AUSLBO). Written informed consent was given by study participants or the next of kin.

### Biomarker analyses

CSF and EDTA plasma samples were collected, aliquoted, and stored at −80°C according to standard procedures. We measured CSF t‐tau, CSF 14‐3‐3 gamma isoform, plasma tau, and plasma NfL as reported.^7^, ^10^ We analysed CSF and plasma beta‐syn using *in‐house* established classic and digital ELISAs, respectively (supplementary methods).^13^, ^14^ All CSF samples were tested by the second‐generation CSF prion Real‐Time Quaking‐Induced Conversion assay (RT‐QuIC).^16^ sCJD participants were positive, whereas non‐prion RPD cases were negative by RT‐QuIC. All biomarkers were analyzed by operators blinded to patients' diagnoses.

### Statistical analysis

We used the Mann–Whitney U or Kruskal–Wallis test (followed by Dunn‐Bonferroni post hoc test) to compare continuous variables. Spearman's correlations and linear regression analyses were used to test the possible associations between variables. Each marker's diagnostic accuracy was calculated using receiver operating characteristic (ROC) analyses. The DeLong test was used to compare the different areas under the curve (AUCs). Univariate and multivariate Cox regression analyses tested the associations between survival and CSF or plasma beta‐syn, and/or known prognostic factors in sCJD.^10^ We performed survival analyses in the whole cohort of sCJD cases and in the most prevalent clinicopathological subtypes. Statistical tests were two‐tailed, and *p*‐values were considered statistically significant at <0.05. Further details are reported in supplementary methods.

## Results

### CSF and plasma beta‐syn levels in the diagnostic groups

Demographic features and biomarker results in the diagnostic groups are shown in Table 1. Associations between demographic variables and biomarkers and among biomarkers are reported in supplementary results.

**Table 1:** Demographic variables and distribution of CSF and plasma biomarkers across sporadic Creutzfeldt–Jakob disease and non‐prion RPDs.

	Sporadic Creutzfeldt–Jakob disease (*n* = 150)	Non‐prion RPD *n* = 106)	*p* value
Age (years) mean ± SD	67.56 ± 9.77	72.15 ± 10.37	<0.001
Female %	48.7	53.8	0.421
Time from onset to sample collection (months) mean ± SD	3.74 ± 3.93	–	
CSF beta‐syn (pg/mL)	4307 (1724–7301)	297 (195–655)	<0.001
Median (IQR)			
Plasma beta‐syn (pg/mL)	104.6 (44.7–186.6)	10.3 (2.8–27.9)	<0.001
Median (IQR)			
CSF t‐tau (pg/mL)	6392 (2343–10828)	619 (384–1268)	<0.001
Median (IQR)			
CSF 14–3‐3 (AU/mL)	69200 (33225–139250)	9695 (6215–18425)	<0.001
Median (IQR)			
Plasma tau (pg/mL)	7.5 (4.1–22.3)	2.7 (1.8–4.7)	<0.001
Median (IQR)			
Plasma NfL (pg/mL)	112.6 (60.4–208.8)	59.2 (27.9–156.3)	<0.001
Median (IQR)			

Beta‐syn, beta‐synuclein; CSF, cerebrospinal fluid; IQR, interquartile range; NfL, neurofilament light chain; RPD, rapidly progressive dementia; SD, standard deviation; t‐tau, total tau protein.

**Figure 1:**
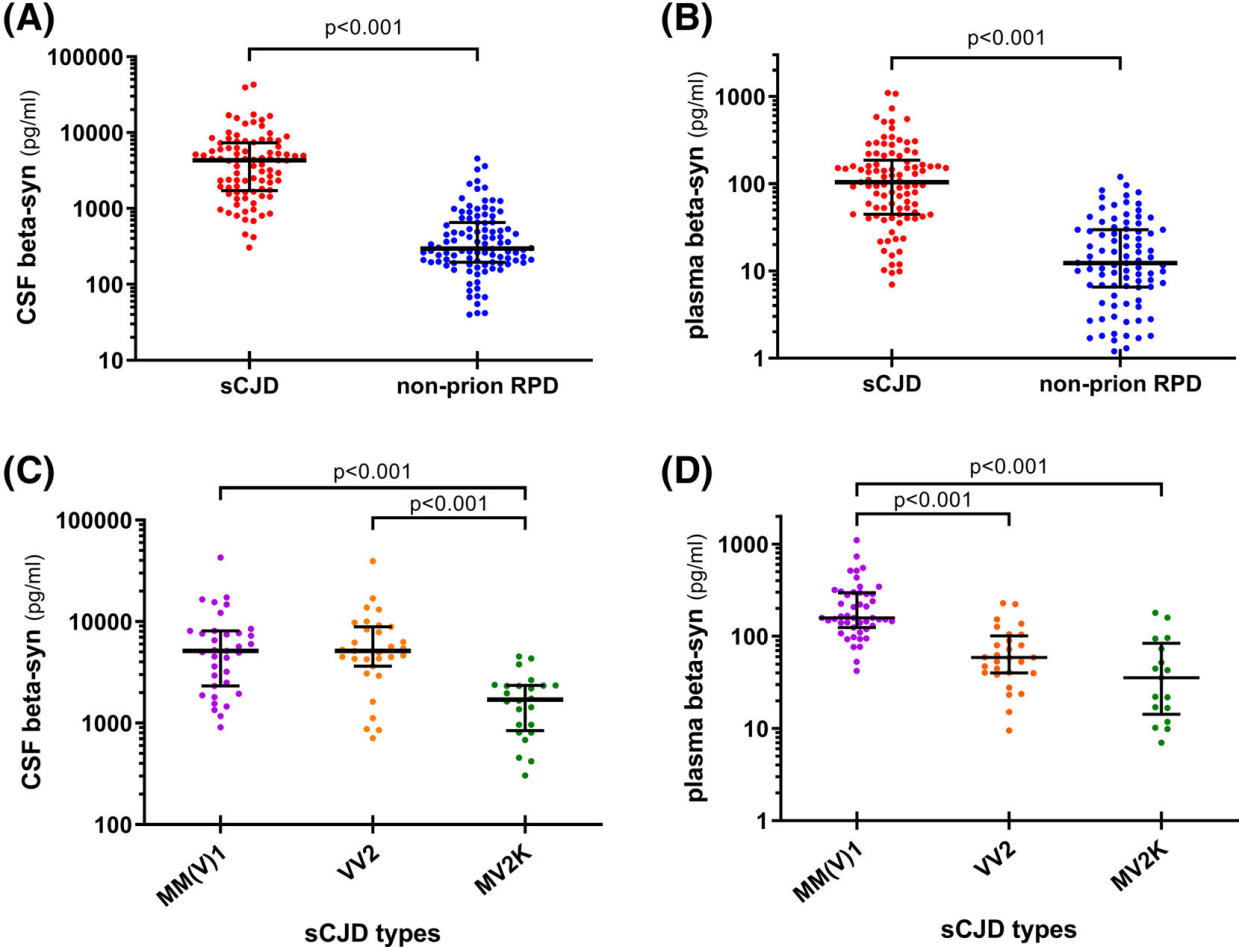
CSF and plasma beta‐syn levels in the diagnostic groups. (A) CSF and (B) plasma beta‐syn values in sporadic Creutzfeldt–Jakob disease and non‐prion RPD; (C) CSF and (D) plasma beta‐syn levels in sporadic Creutzfeldt–Jakob disease most prevalent molecular subtypes [MM(V)1, VV2, and MV2K]. β‐syn values are presented as log(pg/mL); median, and interquartile range are shown for the biomarker levels. *p* values of Mann–Whitney or Dunn's post hoc test after Kruskal–Wallis test are reported. CSF, cerebrospinal fluid; RPD, rapidly progressive dementia; sCJD, sporadic Creutzfeldt–Jakob disease.

sCJD patients showed higher CSF and plasma beta‐syn levels compared to non‐prion RPD subjects (*p* < 0.001 for both) (Fig. 1A,B). Moreover, different CSF and blood beta‐syn distributions were observed for the most prevalent sCJD subtypes (Fig. 1C,D, and Table S2). Both sCJD MM(V)1 and VV2 subgroups demonstrated significantly higher CSF beta‐syn levels compared to the MV2K group (*p* < 0.001 for both comparisons). In plasma, instead, the marker reached the highest values in sCJD MM(V)1 (*p* < 0.001 vs. both VV2 and MV2K) with no significant differences between the VV2 and MV2K groups. All findings mentioned above remained significant after the exclusion of probable cases (supplementary results). The profiles of classic CSF and blood surrogate biomarkers in sCJD subtypes and non‐prion RPD groups are reported in Table 1, supplementary results, Table S3. The distributions of CSF, plasma beta‐syn, and other biomarker values across non‐prion RPD etiologies are shown in Table 2, supplementary results, and Table S4.

**Table 2:** CSF and blood beta‐syn distribution in non‐prion RPD etiologies.

Diagnostic categories of non‐prion RPD patients	*n*	CSF beta‐syn (pg/mL) Median (IQR)	*n*	Plasma beta‐syn (pg/mL) Median (IQR)
All neurodegenerative diseases	52	268 (184–454)	50	10.8 (3.3–25.9)
Alzheimer's disease	42	281 (200–467)	40	11.6 (3.0–26.8)
Dementia with Lewy bodies	8	131.5 (71.5–252.5)	8	8.7 (3.1–18.7)
Frontotemporal dementia	2	243, 82	2	9.4, 36.2
Vascular/mixed dementia and stroke	10	201 (76.5–629)	10	19.6 (1.5–65.4)
Immuno‐mediated, infectious encephalitis and other inflammatory/infective diseases	27	493 (221–1284)	25	7.3 (1.4–34.2)
Toxic/metabolic encephalopathies	10	521 (193–952)	10	13.2 (5.3–30.0)
Central nervous system malignancy	5	659 (387–2772)	3	2.8, 4.0, 0.8

Beta‐syn, beta‐synuclein; CSF, cerebrospinal fluid; IQR, interquartile range; RPD, rapidly progressive dementia.

### Diagnostic and prognostic value of CSF and blood beta‐syn

The ROC curve data of all CSF and blood biomarkers in the differential diagnosis between sCJD and non‐prion RPD are reported in Table 3. Specifically, the discriminative performances of CSF (AUC 0.949 ± 0.014) and plasma beta‐syn (AUC 0.914 ± 0.019) were the highest observed for their respective compartments. At Youden index calculated cutoffs (Table 3), the positive predictive values of CSF and plasma beta‐syn were 77.8% and 83.8%, respectively, whereas the negative predictive values were 96.3% and 84.8%, respectively. Furthermore, CSF beta‐syn performed better than CSF 14‐3‐3 (AUC 0.892 ± 0.022) and, to a lesser extent, than CSF t‐tau (AUC 0.922 ± 0.017). Moreover, the diagnostic value of plasma beta‐syn was higher than that of plasma tau (AUC 0.791 ± 0.032) and plasma NfL (AUC 0.654 ± 0.040), and comparable to that of classic CSF surrogate markers.

**Table 3:** Diagnostic accuracy of CSF and plasma beta‐syn in comparison with that of other CSF and blood biomarkers in the discrimination between sporadic Creutzfeldt‐Jakob disease and non‐prion RPDs.

	AUC		Cutoff	Sens (%)	Spec (%)	DeLong *p* vs. CSF beta‐syn	DeLong *p* vs. plasma beta‐syn
CSF beta‐syn	0.949 ± 0.014	>	1313 pg/mL	84.4	92.2	–	0.135
Plasma beta‐syn	0.914 ± 0.019	>	37.5 pg/mL	84.7	84.0	0.135	–
CSF t‐tau	0.922 ± 0.017	>	1770 pg/mL	86.0	87.7	0.212	0.765
CSF 14‐3‐3	0.892 ± 0.013	>	16550 AU/mL	94.0	73.6	0.028	0.447
Plasma tau	0.791 ± 0.032	>	4.1 pg/mL	77.3	69.7	<0.001	0.001
Plasma NfL	0.654 ± 0.040	>	50.2 pg/mL	88.0	47.5	<0.001	<0.001

AUC, area under the curve; beta‐syn, beta‐synuclein; CSF, cerebrospinal fluid; NfL, neurofilament light chain; sens, sensitivity, spec, specificity; t‐tau, total tau protein.

At cut‐off values favouring sensitivity (99%) over specificity, as in the context of a screening test (Table S5), CSF and plasma beta‐syn showed the highest specificities (60.8% and 46.0%, respectively) compared to the other biomarkers.

The ROC curves for the distinction between sCJD and major non‐prion RPD etiologies are shown in Table S6 and confirm the same results.

Overall, CSF and plasma beta‐syn concentrations were significantly associated with survival in sCJD subjects at univariate and multivariate analyses after accounting for age, disease duration (time from onset to sampling), and *PRNP* codon 129 genotype (Table S7). However, the association did not remain significant after adjustment for the clinicopathological subtype nor in the sub‐analysis considering a single molecular subgroup (Table S7).

## Discussion

Beta‐syn is emerging as a promising CSF and blood biomarker for synaptic damage in several neurodegenerative diseases.^12^, ^13^, ^14^ In the present study, we reported higher CSF and plasma beta‐syn levels in sCJD compared to non‐prion RPDs. In prion diseases, the massive synaptic degeneration might lead to beta‐syn release from the presynaptic terminals into the extracellular space and then in biofluids. The finding of the decreased beta‐syn levels in the brain tissue of sCJD patients^14^ and the marked increase in CSF and blood supports this hypothesis. Compared to other synaptic markers, which show extracerebral synthesis (alpha‐synuclein) or lack a sensitive blood assay [neurogranin and synaptosomal‐associated protein 25 (SNAP‐25)],^14^, ^17^, ^18^ beta‐syn is CNS‐specific^19^ and can be reliably measured in blood using a digital ELISA.^14^

As reported for tau protein,^10^ CSF beta‐syn showed similar levels in sCJD MM(V)1 and VV2, whereas plasma concentrations were higher in the former group than in the latter. To explain this behaviour, we previously hypothesized^10^ that, due to the early cortical neuronal and synaptic damage, the brains of subjects with MM(V)1 manifest a higher spillover of neuronal markers in the blood. Indeed, the VV2 subtype shows a predominant subcortical pathology with only late cerebral cortex involvement.^5^, ^10^, ^20^

Most interestingly, CSF beta‐syn performed better than CSF 14‐3‐3 and, to a lesser extent, than CSF t‐tau, and, if adopted as a screening test, correctly excluded a higher number of non‐prion RPDs than all other biomarkers. Thus, while the CSF RT‐QuIC still represents the most sensitive and specific biofluid marker for sCJD, CSF beta‐syn might represent a highly performant first‐level test in patients with suspected sCJD for those laboratories performing the prion seeding assay as a confirmative test when other tests are suggestive of prion disease.^4^, ^7^, ^10^

Nevertheless, the significant added value of beta‐syn mainly relies on the maintenance of a high diagnostic performance in the blood. Blood NfL and tau showed poor discriminative performance in the clinical setting of RPDs.^8^, ^10^, ^11^ Conversely, blood beta‐syn might accurately estimate *noninvasively* the degree of synaptic/neuronal damage with relatively low cost and a potential application for screening and monitoring to a broad population.

In the context of prognostication, CSF and plasma beta‐syn concentrations, like other CSF and blood markers,^10^ might also represent complementary predictive factors for survival together with other variables (i.e., age, *PRNP* codon 129 genotype, etc.). Here, the lack of associations between the biomarker values and survival in the molecular subtypes might partially rely on the small sample size of each subgroup. Moreover, another potential limitation of the study is the lack of clinical measurements of functional status and progression rate, which previously correlated with biomarker values.^9^, ^11^

In conclusion, by providing evidence that the diagnostic value of plasma beta‐syn is comparable to that of classic CSF surrogate markers (i.e., t‐tau and 14‐3‐3), the present results suggest that beta‐syn might represent the first accurate blood marker for the diagnostic screening of patients with suspected sporadic Creutzfeldt–Jakob disease *in vivo*. Further, the marker might contribute to patient stratification and prognostication.

## Author Contributions

Conception and design of the study: Samir Abu‐Rumeileh, Markus Otto, and Piero Parchi; acquisition and analysis of data: Samir Abu‐Rumeileh, Steffen Halbgebauer, Giuseppe Mario Bentivenga, Lorenzo Barba, Simone Baiardi, Andrea Mastrangelo, Patrick Oeckl, Petra Steinacker, Angela Mammana, Sabina Capellari, Markus Otto, and Piero Parchi; drafting of the manuscript: Samir Abu‐Rumeileh, Markus Otto, and Piero Parchi.

## Conflict of Interest Statement

M.O. gave scientific advice to Axon, Biogen Idec, Fujirebio and Roche, all unrelated to the work presented in this paper. The foundation of the state Baden–Wuerttemberg handed in a patent for the measurement of β‐synuclein in neurological diseases. Relevant authors are M.O., S.H., and P.O. The other authors report no conflicts of interest.

## Supporting information


**Data S1** Supporting Information.

## References

[1] Hermann P , Appleby B , Brandel JP , et al. Biomarkers and diagnostic guidelines for sporadic Creutzfeldt‐Jakob disease. Lancet Neurol. 2021;20(3):235‐246. doi:10.1016/S1474-4422(20)30477-4 33609480 PMC8285036

[2] Rudge P , Hyare H , Green A , Collinge J , Mead S . Imaging and CSF analyses effectively distinguish CJD from its mimics. J Neurol Neurosurg Psychiatry. 2018;89(5):461‐466. doi:10.1136/jnnp-2017-316853 29142140 PMC5909756

[3] Watson N , Hermann P , Ladogana A , et al. Validation of revised international Creutzfeldt‐Jakob disease surveillance network diagnostic criteria for sporadic Creutzfeldt‐Jakob disease. JAMA Netw Open. 2022;5(1):e2146319. doi:10.1001/jamanetworkopen.2021.46319 35099544 PMC8804913

[4] Mastrangelo A , Mammana A , Baiardi S , et al. Evaluation of the impact of CSF prion RT‐QuIC and amended criteria on the clinical diagnosis of Creutzfeldt‐Jakob disease: a 10‐year study in Italy. J Neurol Neurosurg Psychiatry. 2023;94(2):121‐129. doi:10.1136/jnnp-2022-330153 36428087

[5] Parchi P , Giese A , Capellari S , et al. Classification of sporadic Creutzfeldt‐Jakob disease based on molecular and phenotypic analysis of 300 subjects. Ann Neurol. 1999;46(2):224‐233. 10443888

[6] Hermann P , Zerr I . Rapidly progressive dementias ‐ aetiologies, diagnosis and management. Nat Rev Neurol. 2022;18(6):363‐376. doi:10.1038/s41582-022-00659-0 35508635 PMC9067549

[7] Abu‐Rumeileh S , Baiardi S , Polischi B , et al. Diagnostic value of surrogate CSF biomarkers for Creutzfeldt‐Jakob disease in the era of RT‐QuIC. J Neurol. 2019 Dec;266(12):3136‐3143. doi:10.1007/s00415-019-09537-0 31541342

[8] Zerr I , Villar‐Piqué A , Hermann P , et al. Diagnostic and prognostic value of plasma neurofilament light and total‐tau in sporadic Creutzfeldt‐Jakob disease. Alzheimers Res Ther. 2021;13(1):86. doi:10.1186/s13195-021-00815-6 33883011 PMC8059191

[9] Staffaroni AM , Kramer AO , Casey M , et al. Association of blood and cerebrospinal fluid tau level and other biomarkers with survival time in sporadic Creutzfeldt‐Jakob disease. JAMA Neurol. 2019;76(8):969‐977. doi:10.1001/jamaneurol.2019.1071 31058916 PMC6503575

[10] Abu‐Rumeileh S , Baiardi S , Ladogana A , et al. Comparison between plasma and cerebrospinal fluid biomarkers for the early diagnosis and association with survival in prion disease. J Neurol Neurosurg Psychiatry. 2020;91(11):1181‐1188. doi:10.1136/jnnp-2020-323826 32928934

[11] Thompson AGB , Anastasiadis P , Druyeh R , et al. Evaluation of plasma tau and neurofilament light chain biomarkers in a 12‐year clinical cohort of human prion diseases. Mol Psychiatry. 2021;26(10):5955‐5966. doi:10.1038/s41380-021-01045-w 33674752 PMC8758487

[12] Oeckl P , Halbgebauer S , Anderl‐Straub S , et al. Targeted mass spectrometry suggests Beta‐synuclein as synaptic blood marker in Alzheimer's disease. J Proteome Res. 2020;19:1310‐1318. doi:10.1021/acs.jproteome.9b00824 32101007

[13] Halbgebauer S , Oeckl P , Steinacker P , et al. Beta‐synuclein in cerebrospinal fluid as an early diagnostic marker of Alzheimer's disease. J Neurol Neurosurg Psychiatry. 2021;92(4):349‐356. doi:10.1136/jnnp-2020-324306 33380492

[14] Halbgebauer S , Abu‐Rumeileh S , Oeckl P , et al. Blood β‐synuclein and neurofilament light chain during the course of prion disease. Neurology. 2022;98(14):e1434‐e1445. doi:10.1212/WNL.0000000000200002 35110380

[15] Abu‐Rumeileh S , Barschke P , Oeckl P , et al. Prodynorphin and proenkephalin in cerebrospinal fluid of sporadic Creutzfeldt‐Jakob disease. Int J Mol Sci. 2022;23(4):2051. doi:10.3390/ijms23042051 35216166 PMC8877714

[16] Franceschini A , Baiardi S , Hughson AG , et al. High diagnostic value of second generation CSF RT‐QuIC across the wide spectrum of CJD prions. Sci Rep. 2017;7(1):10655. doi:10.1038/s41598-017-10922-w 28878311 PMC5587608

[17] De Vos A , Jacobs D , Struyfs H , et al. C‐terminal neurogranin is increased in cerebrospinal fluid but unchanged in plasma in Alzheimer's disease. Alzheimers Dement. 2015;11(12):1461‐1469. doi:10.1016/j.jalz.2015.05.012 26092348

[18] Halbgebauer S , Steinacker P , Hengge S , et al. CSF levels of SNAP‐25 are increased early in Creutzfeldt‐Jakob and Alzheimer's disease. J Neurol Neurosurg Psychiatry. 2022;93:1059‐1065. doi:10.1136/jnnp-2021-328646 35995553

[19] Uhlén M , Fagerberg L , Hallström BM , et al. Proteomics. Tissue‐based map of the human proteome. Science. 2015;347(6220):1260419. doi:10.1126/science.1260419 25613900

[20] Baiardi S , Magherini A , Capellari S , et al. Towards an early clinical diagnosis of sporadic CJD VV2 (ataxic type). J Neurol Neurosurg Psychiatry. 2017;88(9):764‐772. doi:10.1136/jnnp-2017-315942 28668775

