# Supplementary material for: High diagnostic performance of plasma and cerebrospinal fluid beta‐synuclein for sporadic Creutzfeldt–Jakob disease
Source: Ann Clin Transl Neurol. 2023 Aug 8;10(10):1904–9. doi: 10.1002/acn3.51873 (PMC10578883; doi:10.1002/acn3.51873)
Supplement: Supplementary file 1 — Data S1 Supporting Information. [file ACN3-10-1904-s001.pdf]

SUPPORTING INFORMATION

SUPPLEMENTARY METHODS

Case classification

Inclusion criteria were: i) suspicion of Creutzfeldt-Jakob disease at the time of LP/blood collection, ii) definite (neuropathological) diagnosis or probable clinical diagnosis at follow-up according to the current European diagnostic criteria,[1] and iii) available plasma and/or CSF samples for most analyses. We excluded patients with genetic Creutzfeldt-Jakob disease since we recently described blood beta-syn levels in a broad genetic Creutzfeldt-Jakob disease cohort.[2] Of the 256 cases, 137 had both biofluids available, 55 had only CSF available, and 64 had only plasma available. Definite sporadic Creutzfeldt-Jakob disease cases included 45 MM(V)1, 24 VV2, 11 MV2K, 1 MM2C and 1 VV1 subjects.[3,4]

Two consultant neurologists (SB, and PP) made the classification of probable sporadic Creutzfeldt-Jakob disease subtypes after reviewing typical clinical features, disease duration at death or at last follow-up, the results of codon 129 genotype (MM, MV, and VV), CSF biomarkers, and brain magnetic resonance imaging as described.[5]

Probable sporadic Creutzfeldt-Jakob disease VV2 cases (n=20) were all homozygotes VV at codon 129, presented with early and prominent rapidly progressive early ataxia and had a disease duration < 12 months, showed a positive prion RT-QuIC and had at least 2 of the following: 14-3-3 > 23500 AU/ml and/or t-tau levels > 1250 pg/ml, prominent striatum and/or thalamic involvement at brain MRI in the early phase of the disease.[1,3-9] Patients with features highly suggestive of the VV1 subtype were excluded: age at onset ≤36 years, disease duration ≥17 months, and predominant cortical symptoms/signs without ataxia.[7]

Probable sporadic Creutzfeldt-Jakob disease MV2K (n=23) were all heterozygous MV at codon 129, showed prominent ataxia and/or cognitive decline at onset, a disease duration > 8 months, a positive prion RT-QuIC reaction and typical brain MRI (DWI/FLAIR sequences).[1,3-6,8,9]

A patient (129MV) with disease duration < 8 months and presenting with a multisystemic neurological syndrome was classified as probable MV1 (n=1).[1,3-6,8,9]

Probable sporadic Creutzfeldt-Jakob disease MM2C (n=2) were all homozygous at codon 129, had a prominent cognitive decline at onset, a disease duration > 8 months, and were positive at prion RT-QuIC and at brain MRI (DWI/FLAIR sequences).[1,3-6,8,9]

The probable sCJD MM2T (n=1) participant was homozygote MM at codon 129, showed prominent psychiatric, sleep, and oculomotor disturbances as early clinical signs, disease duration of 10 months, was positive at prion RT-QuIC, showed a significant bilateral thalamic hypometabolism in brain FDG-PET and a severe reduction of total sleep time and/or a disorganized sleep in the video-polysomnography.[10]

Patients (129 MM) with disease duration or reaching akinetic mutism in less than 6 months and presenting with a multisystemic neurological syndrome were classified as probable MM1 (n=21).[1,3-6,8,9]

Patients with Alzheimer's disease (rapidly progressive form) were diagnosed at autopsy (n=4) or according to the international criteria (n=43),[11] including the presence of a characteristic Alzheimer's disease CSF biomarker profile according to our in-house cutoff values.[12] Further, the diagnosis of rpAD required at least one of the following: (1) rapid cognitive decline with or without motor signs leading to the clinical suspicion of prion disease, (2) CSF t-tau > 1100 pg/ml.[13,14]

Table S1. Etiologies of non-prion RPD cases.

**Diagnostic categories of non-prion RPD patients**	**Definitive diagnosis**		
	**All** *n*	**Pathological** *n*	**Clinical/ Biochemical** *n*
**All neurodegenerative diseases**	53	4	49
Alzheimer's disease	43	4	39
Dementia with Lewy bodies	8	0	8
Frontotemporal dementia	2	0	2
**Vascular/mixed dementia and stroke**	11	2	9
**Immuno-mediated, infectious encephalitis and other inflammatory/ infective diseases**	27	4	23
**Toxic/metabolic encephalopathies**	10	1	9
**Central nervous system malignancies**	5	4	1

RPD = rapidly progressive dementia

Biomarker analyses

CSF t-tau was analysed using a commercially available enzyme-linked immunosorbent assay (ELISA) kit (INNOTEST htau-Ag, Innogenetics/Fujirebio Europe, Ghent, Belgium) according to the manufacturer's instructions.[15] The 14-3-3 gamma isoform was measured using a commercially available ELISA assay kit (Circulex 14-3-3 gamma ELISA kit, MBL, Woburn, MA) according to the manufacturer's instructions.[15]
Plasma tau and NfL were measured on the Simoa SR-X analyzer platform (Quanterix, Billerica, Massachusetts, USA) using Simoa Human t-tau and the Simoa NF-light advantage kits, respectively.[16]
Beta-synuclein CSF levels were measured with an *in-house* established sandwich ELISA, as previously described.[17] In brief, Nunc Maxisorp 96-well plates (Thermo Fisher Scientific, Massachusetts, USA) were coated with 100 µL of a capture antibody (EP1646Y, Abcam, Cambridge, UK), diluted in 100 mM bicarbonate–carbonate buffer pH 9.6 at a concentration of 3.3ug/mL. After overnight incubation at +4°C, the coating solution was removed, and the plate was blocked with 320 µL 2% bovine serum albumin (BSA) in phosphate-buffered saline (PBS) solution with 0.05% Tween 20 for 2 hours at room temperature (RT). For the

calibrator preparation, we purchased recombinant beta-synuclein from rPeptide (Watkinsville, Georgia, USA) and determined the exact protein concentration of the stock solution by amino acid analysis (Alphalyse A/S, Odense, Denmark). After removing the blocking buffer, we loaded in each well 100 µL calibrators ranging from 10 to 1000 pg/mL and 100 µL CSF samples diluted in blocking buffer. CSF samples were dilution stable between dilutions of 1:4 up to 1:20. Plates were shaken for 2 min on a small MR1 rocker (Biosan, Riga, Latvia) and then incubated at RT for 1.5 hours without shaking. After sample incubation, each well was washed with 300 µL washing buffer (PBS with 0.05% Tween 20) three times. We used as the detection antibody an anti-beta-synuclein monoclonal antibody purchased from Abcam (EP1537Y, Abcam, Cambridge, UK), biotinylated in a 40:1 ratio according to the biotinylation protocol provided by Thermo Fisher Scientific (Massachusetts, USA). After removing samples and calibrators and washing the plate with 300 µL washing buffer, we added per well 100 µL of previously biotinylated detection antibody at a concentration of 0.66 µg/mL and incubated the plate for 30 min at RT. After a third washing step, 100 µL of a streptavidin-horseradish peroxidase (Vector laboratories, California, USA) solution was added to each well and incubated at RT for 1 hour. Solution was removed, the plate washed and 100 µL 3,3′,5,5′-Tetramethylbenzidin (ThermoFisher Scientific, Massachusetts, USA) were added and incubated for 5.5 min at RT. The reaction was stopped with 100 µL 1M hydrochloric acid per well. Plates were measured at 450 nm and 570 nm reference wavelength. Concentrations were obtained using a 4-parameter standard curve.

Beta-synuclein blood levels were measured with a novel digital ELISA assay.[2] We used a monoclonal antibody specifically recognizing β-synuclein (EP1537Y) as a capture antibody and a monoclonal antibody against α- and β-synuclein (EP1646Y, both Abcam, Cambridge, UK) as a detection antibody. The biotinylation of the detection antibody is described above. The capture antibody was coupled to carboxylated paramagnetic beads (Quanterix) according to the manufacturer's protocol (coating concentration 0.2 mg/mL). A total of 500,000 beads were applied per replicate, of which 75% were Helper Beads (Quanterix). We biotinylated the monoclonal detection antibody with 40× molar excess of biotin and used in a concentration of 0.5 µg/mL. Buffers for beads and detector were phosphate-buffered saline–Tween 0.5% and phosphate-buffered saline–Tween 0.05%, respectively. Streptavidin–β-galactosidase (SBG) (Quanterix) was diluted in SBG buffer (Quanterix) to a final concentration of 150 pM. We used, as a substrate, resorufin β-D-galactopyranoside (Quanterix). Calibrators ranging from 0.625 to 100 pg/mL were prepared with recombinant β-synuclein, and 400 µL of each calibrator was added to a 96-well plate (Quanterix). Plasma samples were diluted 1:2 and shaken for 10 minutes at room temperature at 1,200 rpm. The digital ELISA was run on the Simoa HD-X platform (Quanterix, Billerica, Massachusetts, USA). Diluted sample (220 µL) was added to each well. Beads, detector, SBG, plate, and substrate were placed into the Simoa platform, and a 3-step custom assay was started. The mean intra- and inter-assay coefficients of variation were ≤5% and <15% for all CSF and blood biomarkers.

Statistical analyses

We used IBM SPSS Statistics V.21 (IBM), GraphPad Prism V.7 (GraphPad Software, La Jolla, California, USA), and R software V.4.0.2 (R foundation, Vienna, Austria). Depending on distribution, data were expressed as percentage, mean±standard deviation (SD), or median and interquartile range (IQR). We adopted the χ^2 test for categorical variables. For continuous variables, depending on the data distribution and number of groups, we applied the Mann-Whitney U test, t-test, Kruskal-Wallis test (followed by Dunn-Bonferroni post hoc test) or the ANOVA (followed by Tukey's post hoc test). All reported p values were adjusted for multiple comparisons. We performed multivariate linear regression models to adjust for age the differences in CSF biomarkers between the groups after the transformation of the dependent variable in the natural logarithmic scale. Spearman's correlations and uni- or multivariate regression analyses were performed to test the possible associations between variables. The diagnostic accuracy of each marker was calculated by means of Receiver operating characteristic (ROC) analyses. The optimal cut-off value for each biomarker was defined using the maximized Youden's index. The DeLong was used to compare the different area under the curve (AUC) values. For the analysis of survival, beta-syn concentration was naturally log-transformed to fulfil the normal distribution. Univariate and multivariate Cox regression analyses tested the associations between survival and CSF or plasma beta-syn or known prognostic factors in sporadic Creutzfeldt-Jakob disease such as age at sample collection, disease duration at sample collection, *PRNP* codon 129 genotype and clinicopathological subtype.[16] We performed survival analyses in the whole cohort of sporadic Creutzfeldt-Jakob disease cases and in three separate subgroups, according to the most prevalent clinicopathological subtypes: (1) sporadic Creutzfeldt-Jakob disease MM(V)1, (2) VV2, and (3) MV2K. The results are presented as hazard ratios (HRs) and 95% confidence intervals (CIs). The assumption of proportional hazard was assessed by Schoenfeld residuals. Statistical tests were two-tailed, and p values were considered statistically significant at <0.05.

SUPPLEMENTARY RESULTS

CSF and plasma biomarkers in the diagnostic groups

Patients with non-prion RPD were older than those with sporadic Creutzfeldt-Jakob disease (p<0.001), but there were no differences in sex distribution between the two groups. Age influenced plasma NfL levels in the Creutzfeldt-Jakob disease (r=0.298, p=0.003) and non-prion RPD (r=0.275, p=0.006) groups, but no other biomarker concentrations. At variance, sex showed no effect on blood and CSF biomarkers. Accordingly, all analyses on plasma NfL were adjusted for age.

In the cohort with both CSF and blood samples available, plasma beta-syn correlated moderately with CSF beta-syn (r=0.494, p<0.001). In the whole cohort, CSF beta-syn correlated strongly with CSF t-tau (r=0.875, p<0.001), CSF 14-3-3 (r=0.841, p<0.001), plasma tau (r=0.409, P<0.001) and plasma NfL (r=0.347, p<0.001). We found also good correlations between plasma beta-syn and CSF t-tau (r=0.695, p<0.001), CSF 14-3-3 (r=0.628, p<0.001), plasma tau (r=0.650, p<0.001) and plasma NfL (r=0.444, p<0.001).

CSF tau, CSF 14-3-3, plasma tau, and plasma NfL were higher in sporadic Creutzfeldt-Jakob disease compared to non-prion RPDs ($p<0.001$ for all comparisons; NfL age-adjusted linear regression $\beta=0.232$, $p=0.001$). Comparisons of these biomarkers between molecular subgroups have already been reported.[15,16]

After the exclusion of probable sporadic Creutzfeldt-Jakob disease cases, higher CSF beta-syn levels were found in MM(V)1 ($p=0.004$) and VV2 ($p=0.006$) groups compared to the MV2K group. Further, MM(V)1 subjects showed higher plasma beta-syn values than VV2 ($p=0.001$) and MV2K ($p=0.004$) patients, whereas the level of the biomarker did not differ between VV2 and MV2K groups.

After considering all diagnostic subgroups, there was no significant difference in CSF and blood beta-syn levels among non-prion RPD etiologies. However, when only the two most numerous non-prion RPD etiologies were considered, patients with inflammatory RPD showed higher CSF ($p<0.008$) but not plasma beta-syn concentrations compared to those with neurodegenerative RPD.

Table S2. CSF and blood beta-syn levels in sporadic Creutzfeldt-Jakob disease subtypes.

Subtype	N	CSF beta-syn (pg/ml) Median (IQR)	N	plasma beta-syn (pg/ml) Median (IQR)
**sCJD MM(V)1**	35	5131 (2330-8116)	47	158.3 (124.3-295.8)
**sCJD VV2**	31	5154 (3645-8871)	29	59.0 (40.1-104.6)
**sCJD MV2K**	24	1703 (845-2348)	18	39.3 (15.5-94.9)
**sCJD MM2C**	-	-	3	96.3, 11.9, 44.4
**sCJD MM2T**	-	-	1	40.2
**sCJD VV1**	-	-	1	27.8

Beta-syn = beta-synuclein; CSF = cerebrospinal fluid; IQR = interquartile range; sCJD = sporadic Creutzfeldt-Jakob disease

Table S3. CSF and blood surrogate biomarkers in sporadic Creutzfeldt-Jakob disease subtypes.

Subtype	N	CSF t-tau (pg/ml) Median (IQR)	N	CSF 14-3-3 (AU/ml) Median (IQR)	N	Plasma tau (pg/ml) Median (IQR)	N	Plasma NfL (pg/ml) Median (IQR)
**sCJD MM(V)1**	67	7165 (3165-11500)	67	92300 (40300-140000)	47	20.5 (10.0-36.0)	49	107.0 (66.6-217.0)
**sCJD VV2**	44	10040 (6605-14690)	44	126000 (75050-184000)	29	4.3 (2.8-5.8)	29	127.8 (97.7-250.3)
**sCJD MV2K**	34	1832 (1200-2558)	34	22350 (16675-34250)	17	4.1 (2.3-13.7)	18	64.9 (48.6-140.3)
**sCJD MM2C**	3	6520, 1185, 1134	3	50500, 16600, 11000	3	15.2, 5.2, 9.7	3	77.0, 52.0, 44.0
**sCJD MM2T**	1	352	1	3699	1	4.5	1	49.1
**sCJD VV1**	1	3620	1	53900	1	1.8	1	274.7

CSF = cerebrospinal fluid; IQR = interquartile range; NfL = neurofilament light chain; sCJD = sporadic Creutzfeldt-Jakob disease; t-tau = total tau protein

Table S4. CSF and blood surrogate biomarkers in non-prion RPD etiologies.

Diagnostic categories of non-prion RPD patients	N	CSF t-tau (pg/ml) Median (IQR)	N	CSF 14-3-3 (AU/ml) Median (IQR)	N	Plasma tau (pg/ml) Median (IQR)	N	Plasma NfL (pg/ml) Median (IQR)
**All neurodegenerative**	53	588 (423-839)	53	8007 (5895-12600)	51	2.6 (1.8-3.8)	52	39.5 (22.2-85.5)
Alzheimer's disease	43	621 (445-991)	43	8818 (6772-12900)	42	2.6 (1.9-3.8)	42	34.9 (20.8-70.7)
Dementia with Lewy bodies	8	310 (240-464)	8	5401 (4046-8387)	7	2.3 (1.4-3.4)	8	55.0 (23.6-183.7)
Frontotemporal dementia	2	470, 6242	2	588, 8050	2	5.0, 1.6	2	50.2, 236.4
**Vascular/mixed dementia and stroke**	11	421 (328-1500)	11	10800 (8030-29400)	10	3.8 (1.4-7.5)	10	160.9 (68.1-407.7)
**Immuno-mediated, infectious encephalitis and other inflammatory/ infective diseases**	27	713 (355-1739)	27	12600 (6012-35500)	25	3.3 (1.9-4.7)	25	95.8 (42.2-314.7)
**Toxic/metabolic encephalopathies**	10	755 (502-14441)	10	11750 (6793-27450)	10	3.2 (2.2-7.3)	9	96.0 (44.4-540.3)
**Central nervous system malignancy**	5	681 (338-8718)	5	32600 (7100-133500)	3	6.2	3	82.4

CSF = cerebrospinal fluid; IQR = interquartile range; NfL = neurofilament light chain; RPD = rapidly progressive dementia; sCJD = sporadic Creutzfeldt-Jakob disease; t-tau = total tau protein;

Diagnostic value of CSF and blood beta-syn and associations of beta-synuclein with survival in sCJD

In the ROC analyses, by limiting the analysis to the most frequent and rapidly progressing sporadic Creutzfeldt-Jakob disease subtypes (i.e., MM[V]1 and VV2), the performances of both CSF (AUC 0.974±0.009) and blood beta-syn (AUC 0.952±0.014) increased. Still, the comparisons with other markers remained similar.

Table S5. Specificities of CSF and plasma biomarkers at cut-offs favouring sensitivity (99%) over specificity in the comparison sCJD vs non-prion RPDs.

	AUC		cut-off	spec (%)
CSF beta-syn	0.983±0.008	>	420 pg/ml	60.8
plasma beta-syn	0.921±0.021	>	9.5 pg/ml	46.0
CSF t-tau	0.965±0.011	>	571 pg/ml	45.3
CSF 14-3-3	0.959±0.013	>	7807 AU/ml	36.8
plasma tau	0.821±0.034	>	0.9 pg/ml	3.0
plasma NfL	0.787±0.042	>	30.7 pg/ml	30.3

AUC = area under the curve; beta-syn = beta-synuclein; CSF = cerebrospinal fluid; NfL = neurofilament light chain; RT-QuIC = real-time quaking-induced conversion assay; sCJD = sporadic Creutzfeldt-Jakob disease; sens = sensitivity, spec = specificity; t-tau = total tau protein;

Table S6. Diagnostic accuracies of CSF and blood beta-syn in the differential diagnosis between sporadic Creutzfeldt-Jakob disease and major forms of RPD.

	AUC		cutoff	Sens (%)	Spec (%)	Delong p vs CSF beta-syn	Delong p vs plasma beta-syn
**sCJD vs neurodegenerative RPDs**							
CSF beta-syn	0.983±0.008	>	663 pg/ml	96.7	92.0	-	0.006
plasma beta-syn	0.921±0.021	>	37.2 pg/ml	84.7	88.5	0.006	-
CSF t-tau	0.965±0.011	>	1770 pg/ml	86.0	96.2	0.197	0.064
CSF 14-3-3	0.959±0.013	>	18700 AU/ml	90.0	90.6	0.110	0.120
plasma tau	0.821±0.034	>	4.0 pg/ml	77.3	78.4		0.016
plasma NfL	0.787±0.042	>	50.2 pg/ml	88.0	63.5	<0.001	0.006

	AUC		cutoff	Sens (%)	Spec (%)	Delong p vs CSF beta-syn	Delong p vs plasma beta-syn
**sCJD vs AD**							
CSF beta-syn	0.982±0.009	>	773 pg/ml	94.4	95.0	-	0.010
plasma beta-syn	0.919±0.023	>	33 pg/ml	85.7	88.1	0.010	-
CSF t-tau	0.961±0.013	>	1770 pg/ml	86.0	95.3	0.176	0.109
CSF 14-3-3	0.957±0.014	>	18700 AU/ml	90.0	90.7	0.123	0.153
plasma tau	0.820±0.036	>	4.7 pg/ml	71.1	85.7	<0.001	0.021
plasma NfL	0.817±0.043	>	48.9 pg/ml	89.0	66.7	<0.001	0.042

	AUC		cutoff	Sens (%)	Spec (%)	Delong p vs CSF beta-syn	Delong p vs plasma beta-syn
**sCJD vs inflammatory RPDs**							
CSF beta-syn	0.901±0.031	>	1313 pg/ml	84.4	77.8	-	0.589
plasma beta-syn	0.923±0.026	>	53.2 pg/ml	70.4	96.0	0.589	-
CSF t-tau	0.887±0.034	>	1761 pg/ml	86.0	77.8	0.765	0.407
CSF 14-3-3	0.830±0.047	>	27600 AU/ml	81.3	74.1	0.214	0.088
plasma tau	0.791±0.044	>	4.7 pg/ml	71.1	80.0	0.045	0.011
plasma NfL	0.538±0.076	>	50.1 pg/ml	88.0	32.0	<0.001	<0.001

AUC = area under the curve; beta-syn = beta-synuclein; CSF = cerebrospinal fluid; NfL = neurofilament light chain; RPD = rapidly progressive dementia; RT-QuIC = real-time quaking-induced conversion assay; sCJD = sporadic Creutzfeldt-Jakob disease; sens = sensitivity, spec = specificity; t-tau = total tau protein;

Table S7. Associations of CSF and blood beta-syn with survival time in the whole sporadic Creutzfeldt-Jakob disease cohort and after stratification according to the disease subtype

Diagnostic group	Beta-syn	Univariate Cox regression		Multivariate Cox regression Codon 129-genotype-adjusted*		Multivariate Cox regression Clinicopathological subgroup-adjusted*	
		**HR (95% CI)**	**P**	**HR (95% CI)**	**p**	**HR (95% CI)**	**p**
**Whole sCJD cohort**	CSF (n=89)	1.908 (1.461-2.492)	<0.001	1.432 (1.082-1.894)	0.012	1.288 (0.974-1.703)	0.075
	Plasma (n=93)	1.436 (1.198-1.721)	<0.001	1.349 (1.090-1.670)	0.006	0.939 (0.716-1.232)	0.650
**sCJD MM(V)1**	CSF (n=35)	1.390 (0.908-2.126)	0.129	-	-	-	-
	Plasma (n=47)	0.734 (0.453-1.187)	0.207	-	-	-	-
**sCJD VV2**	CSF (n=30)	1.428 (0.817-2.498)	0.211	-	-	-	-
	Plasma (n=28)	1.415 (0.886-2.262)	0.146	-	-	-	-
**sCJD MV2K**	CSF (n=24)	1.029 (0.578-1.831)	0.923	-	-	-	-
	Plasma (n=14)	0.990 (0.611-1.604)	0.966	-	-	-	-

*Both multivariate Cox regression analyses included age and time from symptoms onset to LP as covariates.
beta-syn = beta-synuclein; CI = confidence interval; CSF = cerebrospinal fluid; HR = hazard ratio

SUPPLEMENTARY REFERENCES

1. Hermann P, Appleby B, Brandel JP, et al. Biomarkers and diagnostic guidelines for sporadic Creutzfeldt-Jakob disease. Lancet Neurol. 2021;20(3):235-246. doi: 10.1016/S1474-4422(20)30477-4.
2. Halbgebauer S, Abu-Rumeileh S, Oeckl P, et al. Blood β-Synuclein and Neurofilament Light Chain During the Course of Prion Disease. Neurology. 2022;98(14):e1434-e1445. doi: 10.1212/WNL.0000000000200002.
3. Parchi P, Giese A, Capellari S, et al. Classification of sporadic Creutzfeldt-Jakob disease based on molecular and phenotypic analysis of 300 subjects. Ann Neurol. 1999;46:224-233.
4. Parchi P, de Boni L, Saverioni D, et al. Consensus classification of human prion disease histotypes allows reliable identification of molecular subtypes: an inter-rater study among surveillance centres in Europe and USA. Acta Neuropathol. 2012;124:517-29.
5. Mastrangelo A, Mammana A, Baiardi S, et al. Evaluation of the impact of CSF prion RT-QuIC and amended criteria on the clinical diagnosis of Creutzfeldt-Jakob disease: a 10-year study in Italy. J Neurol Neurosurg Psychiatry. 2023;94(2):121-129. doi: 10.1136/jnnp-2022-330153.
6. Zerr I, Kallenberg K, Summers DM, et al. Updated clinical diagnostic criteria for sporadic Creutzfeldt-Jakob disease. Brain. 2009;132: 2659-2668.
7. Baiardi S, Magherini A, Capellari S, et al. Towards an early clinical diagnosis of sporadic CJD VV2 (ataxic type). J Neurol Neurosurg Psychiatry. 2017;88:764-772.
8. Lattanzio F, Abu-Rumeileh S, Franceschini A, et al. Prion-specific and surrogate CSF biomarkers in Creutzfeldt-Jakob disease: diagnostic accuracy in relation to molecular subtypes and analysis of neuropathological correlates of p-tau and Aβ42 levels. Acta Neuropathol 2017;133:559-578.
9. Franceschini A, Baiardi S, Hughson AG, et al. High diagnostic value of second generation CSF RT-QuIC across the wide spectrum of CJD prions. Sci Rep. 2017;7(1):10655. doi: 10.1038/s41598-017-10922-w.
10. Abu-Rumeileh S, Redaelli V, Baiardi S, et al. Sporadic Fatal Insomnia in Europe: Phenotypic Features and Diagnostic Challenges. Ann Neurol. 2018;84(3):347-360. doi: 10.1002/ana.25300.
11. Dubois B, Feldman HH, Jacova C, Hampel H, Molinuevo JL, Blennow K, et al. Advancing research diagnostic criteria for Alzheimer’s disease: the IWG-2 criteria. Lancet Neurol. 2014;13(6):614–29. https://doi.org/10.1016/S1474-4422(14)70090-0.
12. Baiardi S, Quadalti C, Mammana A, et al. Diagnostic value of plasma p-tau181, NfL, and GFAP in a clinical setting cohort of prevalent neurodegenerative dementias. Alzheimers Res Ther. 2022;14(1):153. doi: 10.1186/s13195-022-01093-6.
13. Abu-Rumeileh S, Capellari S, Parchi P. Rapidly Progressive Alzheimer's Disease: Contributions to Clinical-Pathological Definition and Diagnosis. J Alzheimers Dis. 2018;63(3):887-897. doi: 10.3233/JAD-171181.

14. Abu-Rumeileh S, Capellari S, Stanzani-Maserati M, et al. The CSF neurofilament light signature in rapidly progressive neurodegenerative dementias. Alzheimers Res Ther. 2018;10(1):3. doi: 10.1186/s13195-017-0331-1.
15. Abu-Rumeileh S, Baiardi S, Polischi B, et al. Diagnostic value of surrogate CSF biomarkers for Creutzfeldt-Jakob disease in the era of RT-QuIC. J Neurol. 2019;266(12):3136-3143. doi: 10.1007/s00415-019-09537-0.
16. Abu-Rumeileh S, Baiardi S, Ladogana A, et al. Comparison between plasma and cerebrospinal fluid biomarkers for the early diagnosis and association with survival in prion disease. J Neurol Neurosurg Psychiatry. 2020;91(11):1181-1188. doi: 10.1136/jnnp-2020-323826.
17. Halbgebauer S, Oeckl P, Steinacker P, et al. Beta-synuclein in cerebrospinal fluid as an early diagnostic marker of Alzheimer's disease. J Neurol Neurosurg Psychiatry. 2021;92(4):349-356. doi: 10.1136/jnnp-2020-324306.
